# Fermented Kamut Wheat Diet Prevents DSS-Induced Colitis via Modulating Gut Microbiota in Mice

**DOI:** 10.3390/ijms26073017

**Published:** 2025-03-26

**Authors:** Juni Lee, Bum Ju Kil, Yeojin Choi, Hyungyung Chai, Donghoon Lee, Hee-Geun Jo, Donghun Lee

**Affiliations:** 1 Department of Herbal Pharmacology, College of Korean Medicine, Gachon University, 1342 Seongnamdae-ro, Sujeong-gu, Seongnam-si 13120, Republic of Korea; 2 Research Institute, MediCRO Co., Ltd., Dongan-gu, Anyang-si 14067, Republic of Korea; 3 GrainOn Co., Ltd., 185 Donggwang-ro, Seocho-gu, Seoul 06580, Republic of Korea; 4 Naturalis Inc., 6, Daewangpangyo-ro, Bundang-gu, Seongnam-si 13549, Republic of Korea

**Keywords:** inflammatory bowel disease, gut microbiome, anti-inflammatory effect, fermented Kamut wheat

## Abstract

Inflammatory bowel disease (IBD) is a chronic and relapsing inflammatory disorder of the gastrointestinal tract with limited treatment options. This study investigates the preventive effects of fermented Kamut wheat enzyme (FKW) diet on the progression of dextran sulfate sodium (DSS)-induced colitis in mice, with a focus on gut microbiota modulation and inflammatory cytokine regulation. Female C57BL/6J mice were divided into groups and fed a diet consisting of either a FKW diet (containing 39.80% FKW) or a control diet under 1.25% and 2.50% DSS conditions. The FKW diet was formulated based on the AIN-93G standard rodent formula, with the FKW diet providing comparable amounts of total proteins, crude lipids, and dietary fibers as the control diet. The FKW diet effectively mitigated the progression of colitis, as evidenced by improvements in key indicators such as dietary intake, body weight, colon length, stool consistency, and bleeding, particularly in the 1.25% DSS group. Histopathological analysis revealed preservation of colonic architecture and reduced mucosal damage in the FKW group. The diet also resulted in a significant reduction in pro-inflammatory cytokines (TNF-α, IL-6, IL-1β, and IFN-γ) and myeloperoxidase (MPO) levels, coupled with an increase in anti-inflammatory IL-10. Gut microbiota analysis showed increased abundance of beneficial bacteria such as *Muribaculaceae*, *Lachnospiraceae* NK4A136 and *Bacteroides acidifaciens* and decreased pathogenic bacteria like *Escherichia*/*Shigella* and *Bilophila*. These findings underscore the potential of FKW as a preventive dietary intervention for mitigating the progression of colitis, emphasizing the role of gut microbiota in supporting intestinal health. These results highlight FKW’s potential to reduce the risk of colitis development, providing a foundation for future research into its preventive applications.

## 1. Introduction

Inflammatory bowel disease (IBD) is a term that collectively refers to intestinal diseases, including Crohn’s disease (CD) and ulcerative colitis (UC), which are characterized by chronic inflammation of the small and large intestines, leading to intestinal damage [1]. A diet high in processed foods and low in dietary fiber negatively affects gut microbiota balance, accelerating inflammatory responses and worsening the prognosis of IBD [2,3]. The gut microbiota plays a crucial role in regulating the immune system and maintaining the integrity of the intestinal barrier [4]. Additionally, the metabolic products they generate, such as short chain fatty acids (SCFAs), are essential in modulating inflammatory responses [5]. Therefore, restoring gut microbiota balance is a critical factor in maintaining intestinal health.

Existing pharmacological treatments for IBD often have side effects and are not suitable for preventive measures [6]. Main treatments for IBD include anti-tumor necrosis factor (TNF) agents and immunomodulators, which improve IBD by suppressing inflammatory responses and tissue damage. While there have been reports that these drugs influence the regulation of gut microbiota, non-responders to these medications do not experience an improvement in gut microbiota imbalances, and there are still insufficient cases where key SCFA-producing taxa, critical for regulating intestinal inflammatory responses, have been significantly expanded [7]. Additionally, anti-TNF agents have shown resistance in over 30% of patients, and long-term use of immunomodulators may increase the risk of opportunistic infections due to side effects such as systemic immunosuppression [8,9]. Due to these limitations and risks of current drug treatments, there is a growing preference for managing IBD through foods that can regulate gut microbiota while being safe for long-term consumption. In particular, foods rich in dietary fiber have the potential to improve gut microbiota imbalances by serving as an energy source for gut microbes, offering a new alternative to manage the progression of IBD [10].

In recent years, ancient wheat varieties have gained attention for their health benefits, particularly in the dietary management of chronic diseases. Varieties such as Kamut, Spelt, Emmer and Einkorn are recognized for their rich fiber content, antioxidant-rich phytochemicals and higher bioavailable protein [11]. Of these, Kamut wheat (*Triticum turgidum* subsp. *turanicum*) has been extensively studied for its potential as a nutraceutical [12]. Kamut wheat contains various nutrients, including fiber, amino acids, polyphenols, and carotenoids [13]. It also contains more than three times the amount of selenium found in brown rice and is rich in soluble fiber. Selenium is well-known for its anti-inflammatory and anti-oxidant properties, which protect the body from oxidative damage [14]. In addition to its anti-oxidant properties, selenium-enriched diets have been shown to influence the composition of the gut microbiota, promoting beneficial bacteria [15]. Building on these findings, studies have shown that Kamut wheat reduces oxidative stress and inflammatory markers more effectively than modern wheat varieties, suggesting its potential to extend benefits to gut health [16]. The dietary fiber in Kamut wheat has a prebiotic effect, promoting the growth of beneficial bacteria such as *Lactobacillus plantarum* L12 and *Bifidobacterium pseudocatenulatum* B7003 [17]. Kamut, which is rich in soluble dietary fiber, offers benefits for fermentation [18]. Research indicates that the nutrients present in fermented grains are favorable for the growth and metabolism of microbial communities and help regulate inflammatory responses [19]. Nevertheless, further research is needed to fully understand the specific functions of fermented Kamut on colitis.

In this study, we identified the potential preventive effects of fermented Kamut against the progression of colitis by improving gut microbiota imbalances. A mouse colitis model was created using dextran sulfate sodium (DSS) to induce inflammation at varying severity levels. A 2.50% DSS model was selected to induce a severe colitis model, which is commonly used to study colitis progression and inflammatory responses [20,21]. In contrast, a 1.25% DSS model was used to establish a low-intensity model, allowing for the evaluation of dietary interventions such as fermented Kamut under conditions that permit prolonged consumption. According to previous studies, DSS concentrations above 2% exacerbate gut microbiota dysbiosis, whereas 1–1.5% DSS induces relatively moderate dysbiosis, making it suitable for dietary intervention studies [22]. By analyzing colitis symptoms, cytokine production, and changes in gut microbiota in the mice, we were able to elucidate the mechanism by which fermented Kamut prevents colitis onset and reduces inflammation severity. This provides insight into the role of fermented Kamut and offers a theoretical basis for its application in the prevention of colitis.

## 2. Results

### 2.1. FKW Diet Helped Manage Colitis Symptoms in Mice Compared to Control Diet

**Figure 1:**
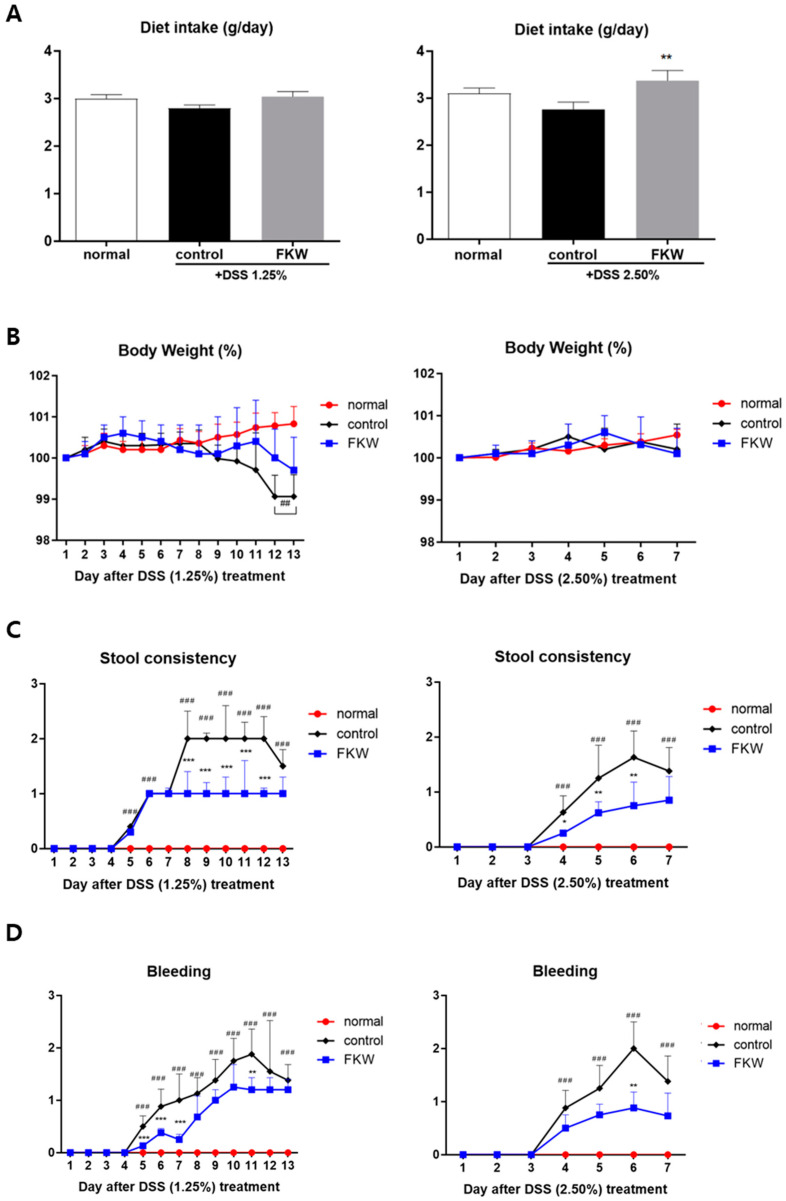
Effects of FKW diet on dietary intake and body weight in DSS-colitis mice. Dietary intake (**A**), body weight (**B**), stool consistency score (**C**) and bleeding score (**D**) of C57BL/6J mice. For the DSS 1.25% groups, C57BL/6J mice were fed 1.25% DSS for 13 days followed by 3 days of water for recovery. For the DSS 2.50% groups, C57BL/6J mice were fed 2.50% DSS for 7 days followed by 5 days of water for recovery. The number of mice was 8 per group. ## *p* < 0.01 vs. normal, ### *p* < 0.001 vs. normal, * *p* < 0.05 vs. control, ** *p* < 0.01 vs. control, and *** *p* < 0.001 vs. control by one-way ANOVA. Effect sizes (η^2^): 0.115 (diet intake, DSS 1.25%), 0.271 (diet intake, DSS 2.50%).

In both control groups (DSS 1.25% and 2.50%), symptoms of intestinal inflammation were observed, including decreased dietary intake, reduced body weight, and increased stool consistency and bleeding scores compared to the normal group (Figure 1A–D). Dietary intake was measured in g per day across all groups. In the DSS 1.25% treatment groups, there were no significant differences in diet intake between the control and FKW groups. However, in the DSS 2.50% treatment groups, the FKW group showed a significant increase in dietary intake compared to the control group (Figure 1A).

Body weight changes were monitored throughout the experimental period. In the DSS 1.25% treatment groups, the control group exhibited a significant decrease in body weight by the 12th and 13th days compared to the normal group. This weight loss is considered to reflect the effects of prolonged intestinal inflammation, impaired nutrient absorption, and disease progression. In contrast, the FKW group maintained a higher body weight than the control group without experiencing significant weight loss, indicating a protective effect of the FKW diet against DSS-induced weight loss. In the DSS 2.50% treatment groups, the normal and FKW groups maintained relatively stable body weights throughout the experimental period, while the control group showed slight fluctuations. This is considered to be due to the relatively short colitis induction period in the DSS 2.50% group, resulting in less pronounced changes in body weight. Although there appears to be a trend towards less weight loss in the FKW group compared to the control group, the difference was not statistically significant (*p* = 0.06, Figure 1B).

Stool consistency scores were recorded daily to evaluate the progression of colitis symptoms. In the DSS 1.25% treatment groups, stool consistency significantly worsened in the control group compared to the normal group, peaking on days 8–12 (### *p* < 0.001). In contrast, the FKW group exhibited significantly lower stool consistency scores compared to the control group during this period (*** *p* < 0.001, Figure 1C). Similarly, in the DSS 2.50% treatment groups, the control group displayed rapid deterioration in stool consistency by day 6, while the FKW group showed significantly better stool consistency scores (** *p* < 0.01).

Bleeding scores were also assessed to evaluate the severity of fecal blood presence. In the DSS 1.25% treatment groups, the control group exhibited significantly increased bleeding scores, particularly on days 5–11 (### *p* < 0.001). In contrast, the FKW group demonstrated significantly lower bleeding scores compared to the control group (** *p* < 0.01, *** *p* < 0.001, Figure 1D). A similar pattern was observed in the DSS 2.50% treatment groups, where the FKW diet significantly reduced bleeding scores compared to the control group during days 4–7 (** *p* < 0.01).

### 2.2. FKW Diet Positively Impacted Colon Length, Spleen Weight, and Histological Score in Mice Compared to Control Diet

**Figure 2:**
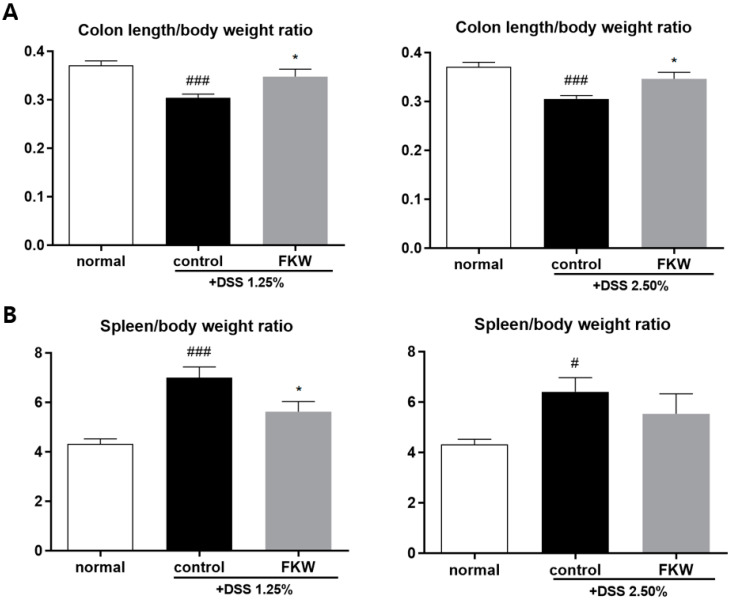
Effects of FKW diet on colon length and spleen weight in DSS-colitis mice. Colon length-to-body weight ratio (**A**) and spleen to body weight ratio (**B**) of C57BL/6J mice. The number of mice was 8 per group; # *p* < 0.05 vs. normal, ### *p* < 0.001 vs. normal, and * *p* < 0.05 vs. control by one-way ANOVA. Effect sizes (η^2^): 0.472 (colon, DSS 1.25%), 0.513 (colon, DSS 2.50%), 0.566 (spleen, DSS 1.25%), and 0.243 (spleen, DSS 2.50%).

The DSS groups exhibited significant signs of intestinal inflammation, evidenced by a decrease in the colon length-to-body weight ratio and an increase in the spleen weight-to-body weight ratio compared to the normal group (Figure 2A,B). Specifically, the colon length-to-body weight ratio decreased by approximately 23% in the control groups given DSS (1.25% and 2.50%) compared to the normal group. In contrast, the FKW groups showed an approximate 13% increase in the colon length-to-body weight ratio compared to their respective control groups (DSS 1.25% and 2.50%), indicating a protective effect of the FKW diet (Figure 2A). Moreover, the spleen-to-body weight ratio significantly increased in the DSS 1.25% control group compared to the normal group (### *p* < 0.001). However, this increase was significantly attenuated in the FKW group (* *p* < 0.05), suggesting a reduction in spleen enlargement associated with inflammation (Figure 2B). Although the DSS 2.50% group also showed an increase in the spleen-to-body weight ratio (# *p* < 0.05), the FKW diet did not result in a significant reduction in this ratio compared to the control (DSS 2.50%) group (Figure 2B).

**Figure 3:**
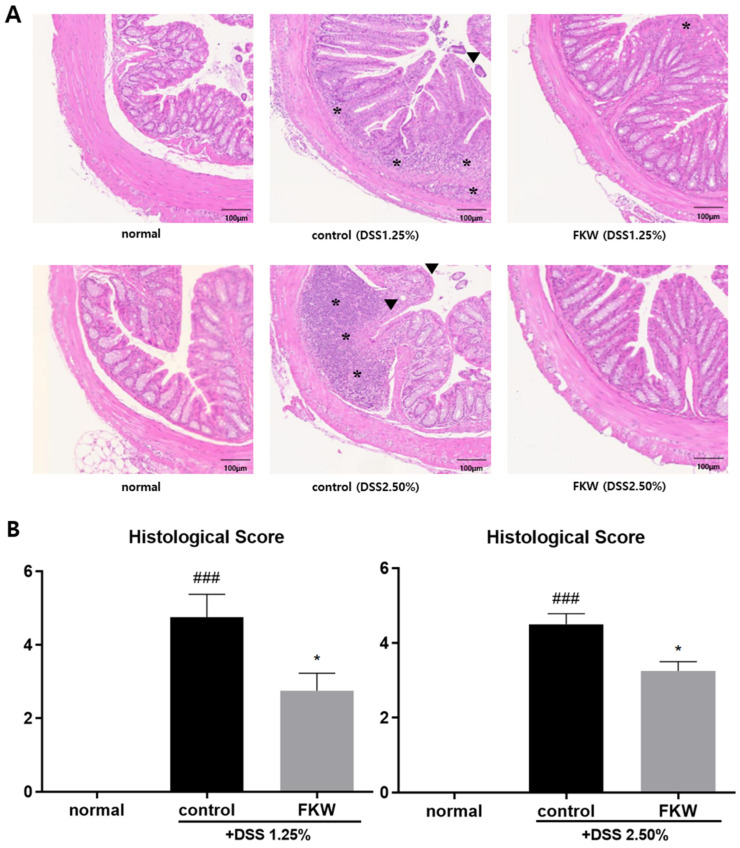
Effects of FKW diet on distal colon in DSS-colitis mice. Histological architecture. Original magnification ×100 (scale bars, 100 μm). (**A**) Histological score; (*) represents inflamed extent, (▼) represents epithelial changes (**B**). The number of mice was 4 per group; ### *p* < 0.001 vs. normal and * *p* < 0.05 vs. control by one-way ANOVA. Effect sizes (η^2^): 0.858 (DSS 1.25%), 0.961 (DSS 2.50%).

In the histological analysis, the DSS group showed that pathology develops severely with the disappearance of crypt structure, goblet cell rarefaction, muscularis mucosa thickening and mucosal immune cell infiltrate. On the other hand, the distal colon in the FKW group showed a relatively intact colonic architecture compared to the control group (Figure 3A). The control groups had the highest scores, while the FKW groups had a lower score than the control group (Figure 3B).

### 2.3. FKW Diet Reduced Inflammatory Markers in Mice Compared to Control Diet

**Figure 4:**
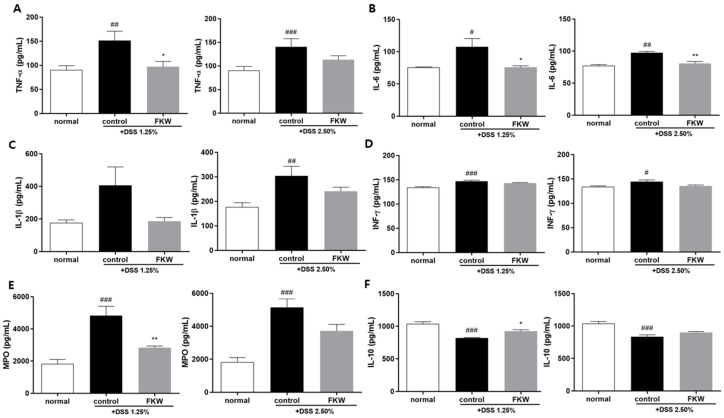
Effects of FKW diet on inflammation biomarkers in DSS-colitis mice. Analysis of proinflammatory cytokines (**A**) TNF-α, (**B**) IL-6, (**C**) IL-1β, (**D**) interferon (INF)-γ, and (**E**) myeloperoxidase (MPO), and (**F**) anti-inflammatory cytokine IL-10 in serum. The number of mice was 6 per group; # *p* < 0.05 vs. normal, ## *p* < 0.01 vs. normal, ### *p* < 0.001 vs. normal, * *p* < 0.01 vs. control and ** *p* < 0.01 vs. control by one-way ANOVA, followed by Tukey’s post hoc test. Effect sizes (η^2^): 0.442 (TNF-α, DSS 1.25%), 0.368 (TNF-α, DSS 2.50%), 0.570 (IL-6, DSS 1.25%), 0.910 (IL-6, DSS 2.50%), 0328 (IL-1β, DSS 1.25%), 0.440 (IL-1β, DSS 2.50%), 0.664 (INF-γ, DSS 1.25%), 0.409 (INF-γ, DSS 2.50%), 0.690 (MPO, DSS 1.25%), 0.690 (MPO, DSS 2.50%), 0.733 (IL-10, DSS 1.25%), 0.663 (IL-10, DSS 2.50%).

Proinflammatory cytokine TNF-α and IL-6 levels in serum were also significantly lower in the FKW group than in the control group (Figure 4A,B). Other inflammatory cytokines (IL-1β, IFN-γ) in the Kamut group also decreased as compared to the control group (Figure 4C–D). Neutrophil-associated molecules (lysosomal protein found in neutrophils), namely MPO, were used as biomarkers to evaluate the severity of intestinal inflammation. MPO showed a significant decrease in FKW groups compared to those in control group (Figure 4E). The anti-inflammatory cytokine IL-10 in serum was significantly increased in the FKW group compared to the control group (Figure 4F). Results showed that FKW reduced the secretion of inflammation biomarker MPO, alleviated the activated TNF-α and IL-6 levels and increased IL-10.

### 2.4. FKW Diet Modulated Gut Microbiota in DSS-Induced Colitis Mice

**Figure 5:**
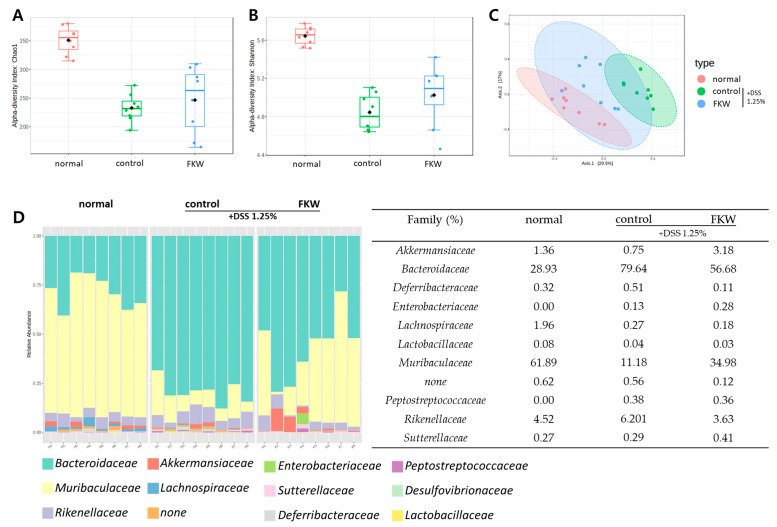
Effects of FKW diet on gut microbiota modulation in DSS-colitis mice. Alpha, beta diversity and bacterial taxa. (**A**) Chao1, (**B**) Shannon, (**C**) Bray–Curtis PCoA plot of beta diversity, (**D**) Relative abundance of bacterial family level. The “none” category in (**D**) represents microorganisms that could not be classified into a specific bacterial family based on 16S rRNA gene analysis. The number of mice was 8 per group.

In this study, we observed that the FKW-1.25% DSS diet group exhibited greater improvement in colitis symptoms compared to the FKW-2.5% DSS diet group, as indicated by inflammation-related markers. To further assess the efficacy of the FKW-1.25% DSS diet, we conducted a comprehensive gut microbiota analysis. Alpha diversity metrics, including Chao1 (F = 22.56, *p* < 0.0001) and Shannon (F = 30.08, *p* < 0.0001), were used to evaluate bacterial diversity using analysis of variance followed by Tukey’s post hoc test. The FKW group showed significant increases in Chao1 and Shannon indices compared to the control group, suggesting an enhancement in bacterial richness and evenness (Figure 5A,B). These indices indicated a notable recovery in microbial diversity within the FKW group, which had been diminished in the control group. Beta diversity was assessed using Bray–Curtis dissimilarities at the genus level. The distinct clustering patterns observed in the beta diversity analysis revealed a clear separation between the normal group and the control group, with the FKW group’s cluster gradually aligning more closely with that of the normal group (Figure 5C). This shift in the FKW group indicates a significant modification of the gut microbiome composition, closer to a healthy state. Relative abundance at the family level was also analyzed (Figure 5D). The FKW group demonstrated an increase in beneficial bacteria such as *Muribaculaceae* and *Lachnospiraceae*, compared to the control group. Specifically, the control group exhibited higher levels of *Bacteroidaceae* and lower levels of *Muribaculaceae* than the normal group.

**Figure 6:**
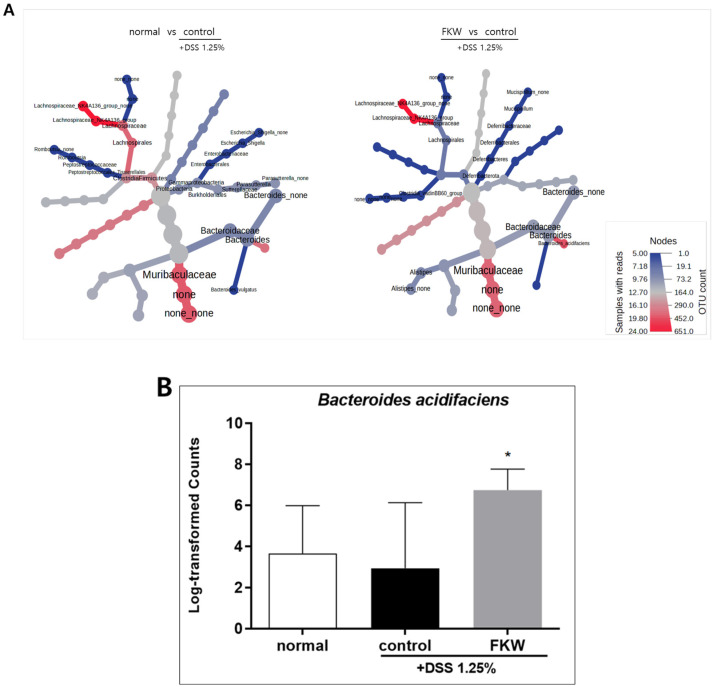
The FKW diet produced similar effects on bacterial taxa in DSS-colitis mice as the normal diet. Heat tree matrices display changes in taxa abundance between groups (**A**), with red nodes indicating increased abundance in the normal and FKW groups compared to the control, and blue nodes indicating the opposite. Log-transformed counts of *Bacteroides acidifaciens* are presented in (**B**). * *p* < 0.05 vs. control by one-way ANOVA. Effect sizes (η^2^): 0.366.

As shown in Figure 6A, the relative abundances of *Muribaculaceae*, *Lachnospiraceae* NK4A136, and *Bacteroides acidifaciens* were higher in both the normal and FKW groups compared to the control group. Conversely, the control group showed increased levels of *Escherichia*/*Shigella*, *Bacteroides*. This pattern was consistently observed when comparing the normal group to the control group and the FKW group to the control group. Specifically, *Bacteroides acidifaciens* levels were significantly higher in the FKW group than in the normal group (Figure 6B).

**Figure 7:**
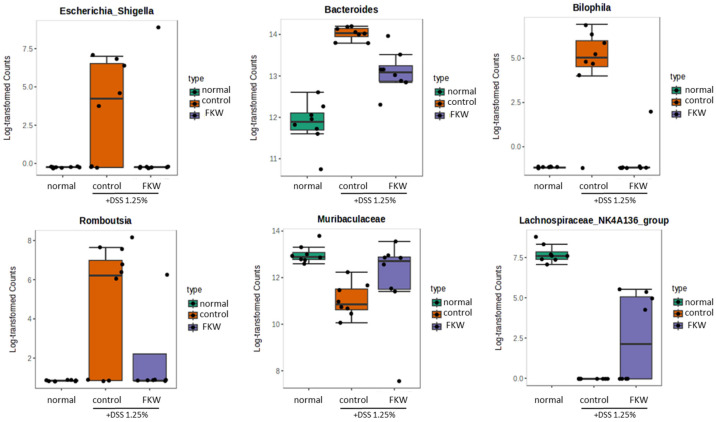
Effects of FKW diet on bacterial taxa and their counts in DSS-colitis mice. The number of mice was 8 per group.

Figure 7 reveals that the log transformed counts of gut microbiota, such as *Escherichia_Shigella*, *Bacteroides*, *Bilophila* and *Romboustia,* were reduced following FKW treatment. *Muribaculaceae*, *Lachnospiraceae*_NK4A136_group were increased compared to the control group.

**Figure 8:**
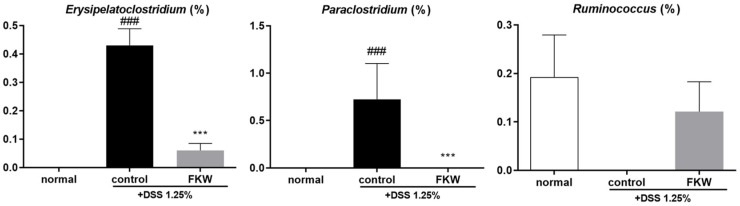
Relative abundance of *Erysipelatoclostridium*, *Paraclostridium* and *Ruminococcus* in experimental groups. The number of mice was 8 per group. ### *p* < 0.001 vs. normal, *** *p* < 0.001 vs. control by one-way ANOVA. Effect sizes (η^2^): 0.788 (*Erysipelatoclostridium*), 0.538 (*Paraclostridium*), 0.192 (*Ruminococcus*).

The relative abundances of *Erysipelatoclostridium*, *Paraclostridium*, and *Ruminococcus* were significantly affected by the FKW diet in DSS-induced colitis mice (Figure 8). For *Erysipelatoclostridium*, the control group exhibited a markedly higher relative abundance compared to the normal group, while the FKW group showed a significant reduction in *Erysipelatoclostridium* abundance compared to the control group. Similarly, *Paraclostridium* levels were significantly elevated in the control group compared to the normal group, but the FKW group demonstrated a substantial decrease in *Paraclostridium* abundance relative to the control group. In the case of *Ruminococcus*, the normal group exhibited a higher relative abundance compared to the control group, which had reduced levels. The FKW group showed a recovery in *Ruminococcus* levels similar to the normal group.

**Figure 9:**
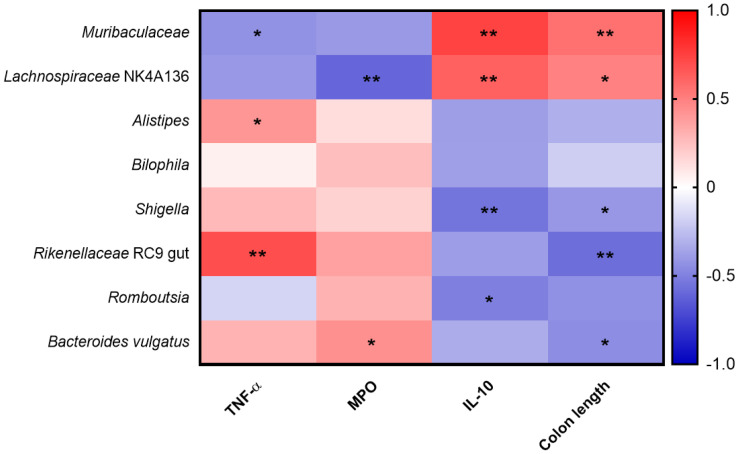
Correlation between gut microbiota and inflammation biomarkers in DSS-induced colitis in the experimental group. Positive correlation is displayed in red, and negative correlations in blue. * *p* < 0.05 and ** *p* < 0.01 by *t*-test.

The heatmap illustrates the correlation between various gut microbiota and inflammatory biomarkers, as well as colon length, in DSS-induced colitis mice treated with the FKW diet. Positive correlations are indicated in red, while negative correlations are shown in blue (Figure 9). *Muribaculaceae* exhibited a strong positive correlation with IL-10 and colon length, while showing a negative correlation with TNF-α. *Lachnospiraceae* NK4A136 demonstrated a positive correlation with IL-10 and colon length, while negatively correlating with MPO. *Alistipes* showed a significant positive correlation with TNF-α. *Bilophila* did not show any significant correlations with the measured markers. *Shigella* exhibited strong negative correlations with IL-10 and colon length, along with a positive correlation with MPO. *Rikenellaceae* RC9 gut content was positively correlated with TNF-α and negatively correlated with colon length. *Romboutsia* showed a negative correlation with IL-10. *Bacteroides vulgatus* demonstrated a significant positive correlation with MPO.

## 3. Discussion

### 3.1. Experimental Approach to Assess the Effects of FKW

This study is the first to demonstrate the potential preventive effects of FKW on colitis progression through an analysis of intestinal length, cytokine production, and gut microbiota composition in a DSS-induced colitis mouse model. The FKW diet reduced the risks associated with pathological changes of colitis, including reduced colon length, high disease scores, and increased inflammatory responses, by modulating gut microbial imbalance. Our study focused on the potential of FKW, rich in dietary fiber, to alleviate inflammation through modulation of the gut microbiota. However, the DSS-induced colitis model severely disrupts the intestinal environment, making it challenging to adequately evaluate the microbiota-regulating effects of FKW. To address this limitation and effectively assess the characteristics of FKW, we adopted a preventive experimental design in which FKW was administered prior to DSS treatment.

### 3.2. FKW May Mitigate DSS-Induced Colonic Damage and Tissue Alterations

The FKW diet prevented inflammatory changes in the DSS mouse model, as demonstrated by the mitigation of colon shortening, improved stool consistency and reduced bleeding scores, and decreased spleen size (Figure 1, Figure 2 and Figure 3). DSS is a substance that induces mucosal inflammation in the colonic lumen [23], closely mimicking the characteristic symptoms of human UC, including mucosal ulcers, weight loss, colon shortening, severe diarrhea, and bloody stools [24]. In patients with a disrupted gut microbiota, the intestinal environment becomes vulnerable to pathogenic invasion, leading to extensive infiltration of inflammatory cells, colonic edema, and ulceration that destroy and shorten the intestinal structure [25]. To assess localized colonic inflammation, analyses of colon length and histological scores were performed. The group consuming the FKW diet exhibited a mitigation of colon shortening, along with improvements in stool consistency scores and bleeding scores [26,27], which are measures of colitis severity. Additionally, the FKW diet helped maintain body weight and food intake. Histological analysis showed that while the control group exhibited extensive crypt distortion and diffuse submucosal inflammatory infiltration, similar to what is observed in human colitis samples, these effects were improved in the FKW diet group. The observed histological damage confirms that DSS-induced colitis was successfully established in the experimental model. This suggests that the FKW diet can attenuate the progression of DSS-induced inflammatory responses and protect the intestinal environment.

### 3.3. FKW May Restore Gut Microbiota Diversity and Protect Against DSS-Induced Dysbiosis

In this study, fecal samples were utilized to comprehensively analyze the gut environment and its microbiota composition. An analysis of the gut microbiota revealed that microbial diversity decreased in the DSS group compared to the normal group, while the FKW group significantly restored this reduced diversity. In the analysis of alpha diversity, which quantitatively evaluates species diversity within a sample, both the Chao1 index [28], which estimates the actual number of species within a sample, and the Shannon index [29], which assesses the evenness of species based on their relative abundance, showed that the FKW group restored both species diversity and evenness compared to the DSS group (Figure 5A,B). Furthermore, the DSS group exhibited specific changes at the family level, with an increase in *Bacteroidaceae* and a decrease in *Muribaculaceae* (Figure 5D). These findings show a similar pattern to the gut microbiota changes observed in IBD patients [30,31]. Given the clear changes at the family level, Bray–Curtis distance was used to further analyze differences in microbial community composition between samples at the species level [32]. The results showed distinct clustering between the normal and DSS groups, with no overlap between their clusters (Figure 5C). In contrast, the FKW diet group showed a shift in clustering from the DSS group toward the normal group. This suggests that the FKW diet may have directly or indirectly influenced the gut microbial community composition, potentially mitigating the dysbiosis induced by DSS.

### 3.4. FKW May Regulate Immune Responses and Gut Microbial Balance to Suppress Intestinal Inflammation

The Stacked bar plot revealed a significant decrease in *Muribaculaceae* in the DSS group, while the FKW group showed a recovery in its abundance (Figure 5D). The hierarchical clustering analysis using the Heat tree matrix demonstrated that the FKW group had higher levels of *Muribaculaceae* and *Lachnospiraceae*_NK4A136_group compared to the DSS group. In IBD, intestinal damage is known to be driven by the release of inflammatory cytokines like INF-γ and TNF-α from overactivated CD8 T cells, which lead to the destruction of the intestinal epithelium and increased gut permeability [33]. Additionally, recent findings suggest that a deficiency in Tregs producing IL-10 contributes to the disruption of intestinal immune balance and increased risk of inflammatory responses [34,35]. Recent studies indicate that *Muribaculaceae* inhibits CD8 T cell activation and reduces excessive immune responses through propionate production [36], while *Lachnospiraceae* promotes Treg cell generation [37]. An increase in Proteobacteria is consistently observed in IBD patients, and this study also confirmed an increase in *Escherichia*/*Shigella* and *Bilophila* in the DSS group. *Escherichia*/*Shigella* was positively correlated with intestinal inflammatory responses [36,38]. *Bilophila* contributes to the exacerbation of colitis by producing LPS, which induces IL-6 and promotes intestinal barrier damage [39,40]. The FKW diet restored the levels of *Muribaculaceae* and *Lachnospiraceae*_NK4A136_group that had been decreased by DSS, while reducing the increased levels of *Escherichia*/*Shigella* and *Bilophila*, thereby improving gut microbial balance. These findings suggest that the FKW diet may suppress immune-mediated inflammatory intestinal damage by modulating gut microbiota.

### 3.5. FKW May Modulate Cytokine Production Through Gut Microbiome Regulation

This study further investigated the correlations between *Muribaculaceae*, *Lachnospiraceae*, *Escherichia*/*Shigella*, *Bilophila*, and inflammatory biomarkers, as well as colon length (Figure 9). *Muribaculaceae* exhibited a negative correlation with TNF-α, a positive correlation with IL-10, and a positive correlation with colon length. These findings align with previous research [38] and support the role of *Muribaculaceae* in reducing TNF-α through the suppression of CD8 T cell activation. The observed increase in *Muribaculaceae* abundance in the FKW group may have contributed to the reduction in TNF-α and the increase in IL-10, which are consistent with its known role in modulating immune responses and maintaining intestinal health. *Lachnospiraceae*_NK4A136_group showed positive correlations with both IL-10 and colon length, likely due to its role in promoting Treg cell generation and enhancing IL-10 levels. Furthermore, the production of butyrate by *Lachnospiraceae* not only promotes intestinal epithelial cell growth, explaining its positive association with colon length, but also exerts anti-inflammatory effects by suppressing pro-inflammatory cytokines, which could explain its negative correlation with MPO. *Lachnospiraceae* exhibited a negative correlation with MPO, potentially linked to its ability to produce Reactive Sulfur Species (RSS), which mitigate oxidative stress and reduce inflammation. This suggests that *Lachnospiraceae* may play a critical role in reducing local inflammation and supporting gut integrity through butyrate production [31,36]. The finding that *Shigella sonnei* induces epithelial inflammation is consistent with the negative correlations between *Shigella*, IL-10, and colon length found in this study. Although some research shows that *Bilophila* affects IL-6 production, this study did not observe significant correlations with TNF-α, MPO, IL-10, or colon length. This lack of correlation may be due to Bilophila’s primary influence on IL-6, which was not analyzed in this study. Through ELISA analysis, we assessed the effects of the FKW diet on serum levels of TNF-α, IL-6, IL-1β, IFN-γ, IL-10, and MPO in animal models to assess the degree of systemic inflammation (Figure 4). The analysis revealed that the FKW group showed a significant reduction in TNF-α and MPO levels, alongside a significant increase in IL-10 production. These results strongly suggest that the modulation of cytokine production is mediated by the increased abundance of *Muribaculaceae* and *Lachnospiraceae*_NK4A136_group, which collectively contribute to anti-inflammatory effects through butyrate production, Treg cell promotion, and CD8 T cell suppression. Thus, these findings indicate that the FKW diet can modulate cytokine production through gut microbiome regulation.

### 3.6. FKW May Enhance Gut Health Through Microbial Balance and Enzymatic Activity

According to recent studies, there is a significant difference in the beta diversity of gut microbiota between groups that consume fermented foods and those that do not consume fermented foods. The microbiota of the fermented food group is particularly associated with *Bacteroides* spp. [41]. One species within *Bacteroides* spp., *Bacteroides acidifaciens*, functions to protect the intestinal mucosa from harmful pathogens by promoting IgA production [42]. While the FKW diet reduced the overall abundance of *Bacteroides*, it restored the levels of *Bacteroides acidifaciens* to above normal levels (Figure 6B). Enzyme-rich foods can improve digestion, reduce pancreatic and gastric secretions, and create a favorable environment for microbial growth, thereby contributing to gut health [43]. The FKW used in this study contained α-amylase and protease, with enzyme activities measured at 614,547 units and 1409 units per 3 g, respectively. These enzymatic activities are likely to have contributed to a more favorable gut environment, potentially supporting improved digestive function and microbial balance. In patients with progressing colitis, a reduction in the diversity of Firmicutes is often observed [4]. The FKW group demonstrated an increase in *Muribaculaceae*, *Lachnospiraceae* NK4A136, both within Firmicutes, while reducing the abundance of *Erysipelatoclostridium* and *Paraclostridium*, which were elevated in the DSS model (Figure 8). Previous studies have shown that consuming *Bacillus subtilis* and *Lactobacillus plantarum* can increase the proportion of Firmicutes [44], which may be related to the increase observed in the FKW group. However, it is important to focus on selectively promoting beneficial bacterial diversity, rather than merely increasing the quantity of Firmicutes, as suggested by earlier findings. This indicates that the effectiveness of fermentation is not solely dependent on the high enzyme activity of the fermentation strains. Therefore, the appropriate consumption of fermented foods is essential for fostering a beneficial gut microbiota composition.

### 3.7. Limitations and Future Perspectives of FKW Research

This study focused on investigating the effects of fermented Kamut; therefore, we set the control group as a non-fermented grain diet rather than non-fermented Kamut or other fermented grains. This approach allowed for the evaluation of the specific contribution of fermentation to the prevention of colitis-related pathologies. However, the absence of a normal group treated only with FKW diet limits the ability to determine whether the observed effects of the FKW diet are selectively associated with DSS-induced inflammation or independent of it. Including such a group in future studies would enable a more comprehensive assessment of the independent effects of FKW diet, clarifying whether its benefits are confined to inflammatory conditions or extend to broader physiological contexts. Furthermore, this study was a preclinical experiment conducted on mice, and the physiological differences and distinct gut microbiota composition between mice and humans limit the direct applicability of the findings to humans. This points to the need for future clinical studies to explore the clinical potential of the FKW diet. Additionally, considering the limited sample size in this study, future research should utilize larger sample sizes to enhance the reliability of the findings and assess the safety and potential benefits of the FKW diet over an extended period. Importantly, future investigations should also aim to disentangle the specific contributions of the fermentation process to the observed outcomes, providing a clearer understanding of the fundamental mechanisms underlying the effects of this diet.

While there are concerns that gluten in wheat grains may trigger immune responses in individuals with gluten sensitivity [45], recent clinical studies have reported that Kamut wheat consumption exhibits beneficial effects for individuals with irritable bowel syndrome and low-grade inflammatory conditions. Consistent with the implications of our findings, Kamut appears to mitigate gut inflammation and support an improvement in the gut microbiota environment. Specifically, clinical studies have shown that Kamut wheat intake significantly reduces symptoms such as abdominal pain, bloating, and fatigue, while improving stool quality and overall quality of life [46]. Although these results support our findings, further clinical trials are warranted to confirm the preventive effects of FKW on the progression of colitis.

## 4. Materials and Methods

### 4.1. Preparation of Fermented Kamut Wheat Enzyme (FKW)

FKW used in this study was provided by GrainOn Co., Ltd. (Kamut^®^ Brand Wheat Fermentation Enzyme; Seoul, Republic of Korea). The fermentation process employed three specific microbial strains, namely *Aspergillus oryzae* isolated from Meju, *Bacillus subtilis* isolated from traditional Korean soybean paste, and *Lactobacillus plantarum* isolated from Kimchi. *Bacillus subtilis* was fermented for 24 h, while *Aspergillus oryzae* and *Lactobacillus plantarum* underwent a 48 h fermentation at temperatures ranging from 30 °C to 40 °C. During fermentation, the actions of the three microbial strains contributed to the production of enzymes, including α-amylase and protease. After cooling down to room temperature, FKW was dried using a spray dryer. Each batch of FKW was blended and packaged after the drying process was completed.

### 4.2. Analysis of Nutritional Composition in FKW

The AOAC method (2023, 22nd edn.) [47] was followed to measure the chemical composition (Table 1). Using techniques outlined by the Ministry of Food and Drug Safety’s Korean Food Standards Codex, the nutritional composition of FKW was examined. After drying the samples, they were heated for three to five hours at 100 °C in an oven to ascertain their water content. The weight loss following heating was used to determine the water content. The fat content was determined using the Soxhlet extraction method, with circulating ether as the solvent. Protein content was calculated using the semimicro-Kjeldahl method, in which total nitrogen was measured and then converted to a percentage using a conversion factor.

### 4.3. Preparation of AIN-93G and FKW Diets

FKW diets were prepared based on the AIN-93G standard rodent formula [48]. The composition of AIN-93G and FKW are shown in the table below (Table 2). The AIN-93G and FKW diets, purchased from Saeron Bio Inc. (Uiwang-si, Gyeonggi-do, Republic of Korea), were formulated to provide equivalent amounts of total proteins, crude lipids, and dietary fibers.

### 4.4. In Vivo Experimental Design

**Figure 10:**
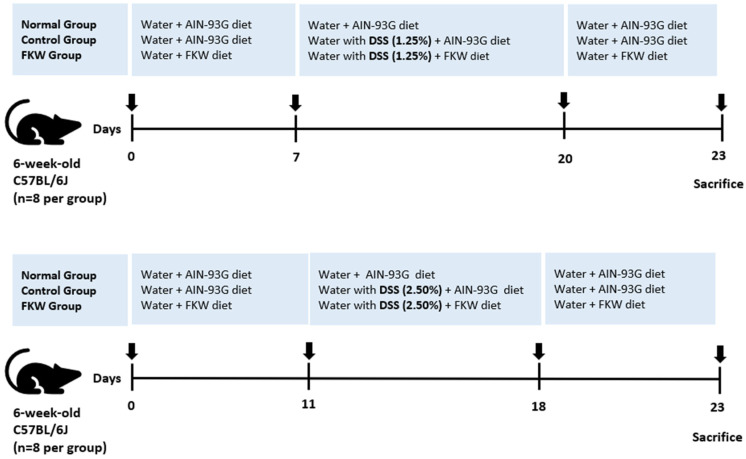
Overview of in vivo experimental design. The experiment consisted of two independent sets: one for the DSS 1.25% group and one for the DSS 2.50% group. Each set included three groups: normal (water and AIN-93G diet), control (DSS with AIN-93G diet), and FKW (DSS with FKW diet). For the DSS 1.25% set, mice were pre-treated with the FKW diet for 7 days prior to DSS administration, followed by 13 days of DSS treatment and 3 days of recovery with water. For the DSS 2.50% set, mice were pre-treated with the FKW diet for 11 days prior to DSS administration, followed by 7 days of DSS treatment and 5 days of recovery with water. The number of mice was 8 per group.

For the in vivo experiment, six-week-old female C57BL/6J mice were obtained from Daehan Bio Link Co., Ltd., Eumseong-gun, Chungcheongbuk-do, Republic of Korea. The mice were submitted to a 7-day acclimation period under normal laboratory conditions, kept on a 12 h day/night cycle at 22 ± 2 °C with a humidity of 45 ± 10%, and allowed to freely access water and food. Inclusion criteria for the study included healthy female mice with a body weight of 18–20 g. Exclusion criteria included animals showing signs of illness or abnormal behavior during the acclimatization period. After acclimation, the mice were then randomly distributed into five experimental groups, with eight mice per group, using randomization software (Excel): normal (water and AIN 93G diet), control-1.25% DSS (AIN 93G diet), FKW-1.25% DSS (FKW diet), control-2.50% DSS (AIN 93G diet), FKW-2.50% DSS (FKW diet). For the 1.25% DSS group, mice were pre-treated with the FKW diet for 7 days prior to DSS administration, and for the 2.50% DSS group, mice were pre-treated with the FKW diet for 11 days prior to DSS administration to investigate its preventive effects on DSS-induced colitis (Figure 10). The sample size of 8 animals per group was chosen based on previous studies in similar experimental models of DSS-induced colitis, where this number was sufficient to detect significant differences in key outcome measures such as inflammatory cytokine levels and histological scores. Additionally, ethical considerations were taken into account to minimize the number of animals used while maintaining statistical reliability. Outcome measures were assessed by investigators who were blinded to the group allocations to ensure objective and unbiased evaluation. After a 1-week stabilization period, the control-1.25% DSS group and the FKW-1.25% DSS group were administered 1.25% DSS (MW 36,000–50,000; MP Biomedicals, Santa Ana, CA, USA) in distilled water, alongside either the AIN-93G diet or the FKW diet, followed by a 3-day recovery period. Similarly, the control-2.50% DSS group and the FKW-2.50% DSS group received 2.50% DSS in distilled water with either the AIN-93G diet or the FKW diet, followed by a 5-day recovery period. All mice were subsequently euthanized using carbon dioxide (CO_2_) asphyxiation. After the mice were sacrificed, their stomachs were opened, and the feces, colon and serum were collected and stored at −80 °C for subsequent analysis.

### 4.5. Assessment of Daily Disease Activity

Each day, dietary intake, stool consistency, body weight, and the presence of total blood in feces were assessed to determine the severity of colitis (Table 3). Stool consistency ratings and the presence of blood in stools were evaluated as previously described [49]. Daily dietary intake was calculated by subtracting the remaining food from the total food provided the previous day (n = 4/cage). Each day, the total amount of food provided was recorded, and the remaining food, including any food retrieved from the cage floor, was measured the following day.

### 4.6. Assessment of Colon Length and Spleen Weight

The colon length and spleen weight ratios relative to body weight were calculated using the following formulas.

Colon length with body weight ratio = colon length (mm)/body weight (g)

Spleen weight with body weight ratio = spleen weight (mg)/body weight (g)

### 4.7. Histological Score in the Distal Colon

The distal colon tissues were fixed in 10% buffered formalin phosphate (Sigma-Aldrich, St. Louis, MO, USA), then embedded in paraffin and stained with hematoxylin and eosin (H&E). The slides were examined under a Nikon SMZ 1270 microscope (Nikon Corporation, Tokyo, Japan) at ×100 magnification, with a scale bar of 100 μm. For the calculation of the histology score, the previously published criteria [26] were amended: inflamed extent (none = 0, mucosa = 1, mucosa and submucosa = 2, and transmural = 3), epithelial changes (none = 0, focal erosions = 1, erosions and focal ulcerations = 2, and extended ulcerations, granulation tissue and pseudopolyps = 3).

### 4.8. Measurement of Cytokine and Myeloperoxidase Level

Blood samples were collected from the heart immediately after euthanasia. The collected samples were immediately placed on ice and after 1 h centrifuged at 2000× *g* for 15 min at 4 °C. The serum was separated and stored at −80 °C until analysis. Enzyme-Linked Immunosorbent Assay (ELISA) kits (TNF-α, IL-6, IL-1β: Proteintech, Wuhan, China; IFN-γ, IL-10: BioLegend, San Diego, CA, USA; myeloperoxidase: Biorbyt, Cambridge, UK) were used to measure serum levels of inflammatory cytokines and myeloperoxidase (MPO), following the manufacturer’s protocol. The absorbance was measured at 450 nm.

### 4.9. Gut Microbiota Analysis

① Sample Collection and DNA Extraction: Fecal samples were collected from experimental mice, immediately frozen in liquid nitrogen, and stored at −80 °C until further processing. Prior to DNA extraction, frozen fecal samples were thawed on ice, and stool samples held in preservation tubes were shaken manually for 10 s. DNA extraction was performed using the QIAamp DNA Stool Mini Kit (Qiagen, Hilden, Germany) following the manufacturer’s protocol. The extracted DNA was quantified using an Epoch™ Spectrophotometer (BioTek, Winooski, VT, USA) to ensure sample quality, following methodologies described in previous studies [27].

② 16S rRNA Sequencing and Data Processing: The bacterial 16S rRNA V3-V4 hypervariable region was amplified using fusion primers 341F (5’-AATGATACGGCGACCACCGAGATCTACAC-XXXXXXXX-TCGTCGGCAGCGTC-AGATGTGTATAAGAGACAG-CCTACGGGNGGCWGCAG-3’) and 805R (5’-CAAGCAGAAGACGGCATACGA-GAT-XXXXXXXX-GTCTCGTGGGCTCGG-AGATGTGTATAGAGACAG-GACTACHVGGGTATCTAATCC-3’). The fusion primers were structured as follows: P5 (P7) graft binding, i5 (i7) index, Nextera consensus, sequencing adaptor, and target region sequence. The final sequence in each primer serves as the target region primer.

Library preparation, including normalization and pooling, was conducted by CJ Bioscience (Seoul, Republic of Korea). Sequencing was performed on the Illumina NovaSeq 6000 platform, generating paired-end reads. Raw reads were demultiplexed and assigned to samples based on unique barcode pairs. The sequence data were processed using DADA2 [50] in QIIME2 for quality filtering, denoising, and chimera removal. Amplicon sequence variants (ASVs) were obtained, and taxonomic classification was performed using the SILVA 138 reference database.

③ Alpha and Beta Diversity Analysis: To assess species richness and evenness within the microbial communities, alpha diversity indices, including the Chao1 index and Shannon index, were calculated. Beta diversity was evaluated using Bray–Curtis distance to compare microbial community structure among different groups. Principal Coordinate Analysis (PCoA) and Non-Metric Multidimensional Scaling (NMDS) were conducted for visualization.

④ Differential Microbiota and Functional Analysis: The composition and relative abundance of bacterial taxa were analyzed at different taxonomic levels using QIIME2, and visualized in bar plots and heat maps. To identify differentially abundant taxa among groups, LEfSe (Linear Discriminant Analysis Effect Size) analysis was performed. Additionally, Random Forest analysis was conducted to classify microbiota differences between groups.

⑤ Correlation and Predictive Functional Analysis: To assess the relationships between gut microbiota and inflammatory biomarkers, Pearson correlation analysis was performed to examine associations between the absolute abundances of key bacterial taxa and cytokine levels, as well as colon length. The Spearman correlation coefficient was also calculated to explore the synergistic or competitive relationships among characteristic bacterial taxa. Functional prediction of microbial communities was performed using PICRUSt2 to infer metabolic pathway activity based on 16S rRNA gene sequences. Spearman correlation analysis was applied to examine associations between microbial metabolic pathways and inflammatory markers. Microbiome data were then subjected to statistical analysis and visualization using MicrobiomeAnalyst 2.0 [51].

### 4.10. Statistical Analysis

Data were reported as mean ± standard error of measurement (SEM) or median with interquartile range. Prior to applying one-way ANOVA and Tukey’s post hoc test, statistical assumptions were verified. Normality was assessed using the Shapiro–Wilk test, and all groups satisfied normality (*p* > 0.05). Homogeneity of variances was tested using the F-test, and the assumption was met (*p* > 0.05). The data were analyzed using one-way ANOVA, followed by Tukey’s post hoc test, under the assumptions of normality and homogeneity of variances. Effect sizes were calculated using eta-squared (η^2^), defined as the ratio of between-group sum of squares (SS_between_) to total sum of squares (SS_total_). Eta-squared was used to evaluate the extent to which DSS treatment and FKW supplementation contributed to the variance in the measured outcomes, providing a measure of effect size for group differences. All statistical analyses, including effect size calculations, were performed using GraphPad Prism software (version 8.01, GraphPad Software, La Jolla, CA, USA) and a statistical package (SPSS PASW Statistics 18, IBM Corp., Hong Kong). *p*-values < 0.05 were considered statistically significant. No data points were excluded from the analysis; all collected data were included in the final dataset.

## 5. Conclusions

The FKW diet modulates gut microbiome composition with preventive effects on colitis-associated symptoms. It helps restore the diversity and balance of gut microbiota, reducing harmful bacteria associated with IBD. Moreover, the FKW diet regulates gut microbiota and influences cytokine production, mitigating inflammatory responses and supporting gut health. Our study highlights that preventive intervention, such as FKW supplementation, plays a significant role in reducing the risk of IBD through gut microbiota modulation and anti-inflammatory mechanisms. This foundational research may guide future clinical studies for preventive applications.

The FKW diet may directly or indirectly modulate the gut microbiome composition, with potential preventive effects on colitis-associated symptoms. Specifically, the FKW diet helps restore the diversity and evenness of gut microbiota disrupted by colitis, increases the proportion of key producers of beneficial metabolites such as SCFAs (propionate and butyrate), and reduces the numbers of harmful bacteria associated with IBD. Moreover, by elucidating the link between intestinal microbiota and markers of inflammation, we confirmed that the FKW diet not only regulates the gut microbiota but also influences cytokine production. Future studies will investigate the host–microbiome interactions based on the metabolic mechanisms of the FKW diet and conduct clinical trials to verify its long-term safety and efficacy in humans. In summary, the FKW diet mitigated inflammatory responses and supported the modulation of microbial imbalances in the gut, providing an innovative perspective for discovering new materials to maintain gut health.

## Figures and Tables

**Table 1:** Nutritional composition of FKW.

Compound	FKW
Carbohydrates (g/100 g)	66.41
Sugars (g/100 g)	14.26
Proteins (g/100 g)	19.16
Selenium (μg/100 g)	112.43

**Table 2:** Ingredients of experimental diets used in this study.

Ingredient (g)	AIN-93G	FKW
Casein	200	134.7
Sucrose	100	100
Dextrose	132	132
Corn Starch	397.5	124.4
Cellulose	50	0
Soybean Oil	70	60.4
Mineral mix	35	35
Vitamin mix	10	10
L-Cystine	3	3
Choline Bitartrate	2.5	2.5
Kamut	-	398
Total	1000.0	1000.0

**Table 3:** Scoring of daily disease activity.

Score	Body Weight Decrease (%)	Stool Consistency	Bleeding
0	<1	Normal	Normal
1	1–5		
2	5–10	Loose stools	
3	10–20		
4	>20	Diarrhea	Gross Bleeding

## Data Availability

All data are contained within the article.
